# Development and Clinical Evaluation of a Large Language Model–Based System for Generating Patient-Friendly Echocardiography Reports: Two-Stage Retrospective Validation and Prospective Survey Study

**DOI:** 10.2196/97136

**Published:** 2026-07-16

**Authors:** Yuanyuan Sun, Chengmin Huang, Huiyuan Kang, Kaimin Wu, Zhiyuan Jin, Caimei Chen, Mulan Liao, Weimei Ou, Bin Wang, Xu Chen, Guoming Zhang

**Affiliations:** 1 Department of Ultrasound, Xiamen Cardiovascular Hospital of Xiamen University, Xiamen, Fujian, China; 2 Emergency Department, Xiamen Cardiovascular Hospital of Xiamen University, Xiamen, Fujian, China; 3 Xiamen Cardiovascular Hospital of Xiamen University, Xiamen, Fujian, China; 4 Emergency Department, Xiamen Chest Pain Quality Control Center, Xiamen Cardiovascular Hospital of Xiamen University, School of Medicine, Fujian Branch of National Clinical Research Center for Cardiovascular Diseases, 2999 Jinshan Road, Xiamen, Fujian, 361009, China, 86 05922293025

**Keywords:** large language model, echocardiography, patient-friendly report, anxiety, digital health, patient-centered care

## Abstract

**Background:**

Standard echocardiography reports use complex terminology, limiting patient comprehension and exacerbating preconsultation anxiety. Large language models (LLMs) can transform technical data into patient-friendly narratives by incorporating longitudinal comparisons with prior examinations.

**Objective:**

This study aims to develop an LLM-based patient-friendly echocardiography reporting system and evaluate its professional safety, patient comprehension, and impact on short-term anxiety.

**Methods:**

This study consisted of 2 stages. In the retrospective development stage, 60 patients were included. Clinical diagnosis, hospitalization records, and serial echocardiographic data were integrated as model inputs using DeepSeek-V3.2. Generated reports followed a standardized 4-module structure. Report quality was independently evaluated by 2 clinicians and an external LLM (Kimi 2.5, Moonshot AI) across 4 domains: data accuracy, information completeness, appropriateness of interpretation, and reasonableness of recommendations. In the prospective clinical evaluation stage, 100 patients undergoing echocardiography and 85 family members were enrolled. Participants received both conventional and LLM-generated patient-friendly reports. A 5-point Likert scale assessed helpfulness in understanding results, effectiveness in addressing concerns, helpfulness in improving disease-related knowledge, and anxiety relief. Anxiety was measured using the STAI-6 (6-item short-form State-Trait Anxiety Inventory) at 3 time points: after echocardiography, after conventional report release, and after reading the patient-friendly report.

**Results:**

All 60 reports in the retrospective stage were successfully generated. Professional evaluation showed high overall quality scores from both clinicians and the external LLM, with no significant difference between evaluators (mean total scores 18.15, SD 1.36 vs 18.28, SD 1.26; *P*=.55). One hallucination event was identified. In the prospective stage, all 100 patients received patient-friendly reports. Both patients and family members rated the reports highly, with no significant between-group difference in total scores (17.61, SD 1.60 vs 17.62, SD 1.03; *P*=.95). Subgroup analyses showed greater perceived benefit among older patients and outpatients (both *P*<.001); these subgroup findings should be considered exploratory given the lack of adjustment for multiple comparisons. Patients with chronic heart failure, reduced left ventricular ejection fraction (≤40%), and left ventricular enlargement (>55 mm) reported higher scores for addressing concerns (all *P*<.001). Anxiety scores increased significantly after conventional report release and decreased significantly after reading the patient-friendly report (both *P*<.001). Older patients (>60 y) and outpatients showed significantly higher anxiety change rates than their counterparts (both *P*<.001). The reduction in anxiety was positively correlated with subjective anxiety relief ratings (*r*=0.531; *P*<.001).

**Conclusions:**

The LLM-based patient-friendly echocardiography reporting system with longitudinal comparison demonstrated good feasibility and promising preliminary clinical usefulness. While maintaining high professional quality, it was associated with improved patient understanding of echocardiographic findings and was associated with reduced short-term anxiety, particularly among older adults and outpatients. Causality cannot be inferred from this nonrandomized sequential design, and longer-term outcomes remain to be evaluated.

## Introduction

Echocardiography is one of the most widely used noninvasive imaging modalities in cardiovascular care, playing a central role in diagnosis, risk stratification, treatment evaluation, and longitudinal follow-up. It provides essential information on cardiac structure, ventricular function, valvular abnormalities, and hemodynamic status. However, standard echocardiography reports are primarily designed for clinicians and are typically dominated by technical terminology, structured measurements, and specialized abbreviations. As a result, patients and family members often struggle to interpret these reports, limiting their ability to engage in informed decision-making and disease self-management [1,2].

This communication gap carries clinical significance. Patients frequently receive their reports before having the opportunity to discuss findings with a physician, and the limited interpretability of conventional reports may contribute to uncertainty, confusion, and anxiety. Prior studies have shown that patients who are unable to understand their test results experience higher levels of distress and lower satisfaction with their care. Moreover, informational needs vary across clinical contexts. Newly referred patients are often most concerned with whether the examination supports a suspected diagnosis, whereas patients with chronic cardiovascular conditions—such as heart failure or post–myocardial infarction remodeling—are more focused on whether their condition has improved, worsened, or remained stable compared with prior examinations [3,4]. Conventional reports, which are organized around a single examination, provide limited support for this type of longitudinal interpretation from the patient perspective.

Recent advances in hospital information systems have made it increasingly feasible to integrate clinical history, diagnostic information, and serial imaging data. At the same time, large language models (LLMs) have demonstrated strong capabilities in information synthesis and natural language generation [5], creating new opportunities to translate complex medical content into patient-centered explanations [6], and the feasibility of using open-source models for more efficient computation and extraction [7]. When combined with structured input design, these models can produce narratives that are both clinically accurate [8] and accessible to lay readers [9,10].

Despite this promise, prior LLM applications in medicine have known risks, including hallucinations, lack of longitudinal integration, and limited prospective validation of patient-centered outcomes. Most existing studies have been retrospective feasibility assessments. Therefore, empirical evidence on safety and real-world psychological impact remains limited. Specifically, cardiovascular care relies heavily on longitudinal monitoring. Patients with chronic heart disease need to know not only their current status but also how it compares to their previous examinations. Most existing LLM prompt engineering frameworks for report simplification focus solely on single, cross-sectional reports, failing to safely integrate and interpret longitudinal clinical history [11,12]. Second, while previous studies have retrospectively validated the clinical safety of LLM-generated reports using physician evaluators, there is a distinct lack of prospective, real-world clinical evidence regarding the actual psychological impact on patients [13]. It remains unknown whether providing an LLM-generated patient-friendly report can effectively mitigate acute anxiety in a real clinical workflow, and whether these benefits vary among different patient demographics, such as older adults or outpatients, who may lack immediate access to medical staff [9,14].

For artificial intelligence (AI) tools, such as LLM-based radiology report simplifiers and patient question-answering systems, a clear distinction should be made between experimental (research-only) systems and those that have been deployed clinically or have received regulatory clearance. We note that most published systems remain at the experimental stage [13,14], and few have been prospectively validated in real-world clinical workflows. This critical perspective helps set appropriate expectations for our own study. To bridge these gaps, we developed an LLM-based digital health intervention system designed to generate patient-friendly echocardiography reports that incorporate longitudinal comparisons. This study was conducted in 2 phases. In phase 1, we retrospectively developed the system and rigorously evaluated its professional safety, accuracy, and hallucination rates using clinician and external LLM reviewers. In phase 2, we conducted a prospective clinical evaluation among patients and their family members in a real-world setting. Our primary objective was to quantitatively assess whether the LLM-generated reports improve comprehension and mitigate acute short-term anxiety (measured by the 6-item short-form State-Trait Anxiety Inventory [STAI-6] scale) triggered by standard medical reports. We hypothesized that this LLM-based intervention would be associated with lower anxiety scores, while acknowledging that causal inference is limited by the nonrandomized design.

## Methods

### Study Design

**Figure 1.**
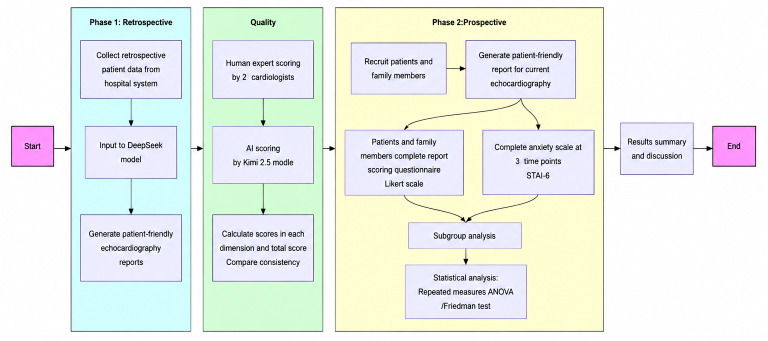
Flowchart of the study design, including the retrospective development and professional evaluation phase and the prospective clinical evaluation phase. AI: artificial intelligence; STAI-6: 6-item short-form State-Trait Anxiety Inventory.

This study adopted a 2-stage, mixed methods design to thoroughly evaluate an LLM-based digital health intervention for echocardiography reporting. Phase 1 comprised the retrospective development of the LLM prompt framework and a rigorous professional evaluation of the generated reports. Phase 2 involved a prospective, real-world clinical survey to assess the impact of the LLM-generated patient-friendly reports on patient comprehension and short-term anxiety. The prospective phase used a sequential pre-post design without randomization or blinding; all participants received the conventional report first, followed by the patient-friendly report, for ethical and practical reasons (conventional reports are the standard of care and withholding them would be unethical; thus, a parallel control group receiving only the patient-friendly report was not feasible). Figure 1 shows the study flowchart.

### Ethical Considerations

The study protocol was reviewed and approved by the Institutional Review Board of Xiamen Cardiovascular Hospital, Xiamen University (2026KY009). For phase 1, patient data were strictly deidentified to ensure privacy. For phase 2, all prospective participants (patients and their family members) provided written informed consent prior to enrollment. Participants were explicitly informed that the LLM-generated reports were for educational and informational purposes only and did not replace professional medical consultation.

### Phase 1: System Development and Retrospective Validation

In phase 1, we randomly selected 60 retrospective cases of patients who had undergone at least 2 echocardiographic examinations (to allow for longitudinal comparison) from our hospital’s database. We developed an LLM-based system for generating patient-friendly longitudinal echocardiography reports using DeepSeek-V3.2 through an application programming interface. To improve clinical relevance and consistency, a structured prompt framework was used. Four categories of information were provided as input: (1) prior medical history, (2) current clinical diagnosis or referral indication, (3) previous echocardiography report or reports for longitudinal comparison, and (4) structured findings and descriptive results from the current examination.

Based on these inputs, the model generated a nontechnical, patient-friendly narrative report using a standardized 4-module format: module 1: interpretation of key findings from the current examination; module 2: comparison with previous echocardiography findings and explanation of interval changes; module 3: plain-language explanation of major abnormalities; and module 4: responses to likely patient concerns with general suggestions based on current cardiac status. The system was designed to process one previous report as the reference and the current examination as the follow-up, enabling a clear longitudinal narrative.

Report quality was independently evaluated by 2 clinicians with experience in cardiovascular ultrasound interpretation and by an external LLM, Kimi 2.5 (Moonshot AI). Kimi 2.5 served as an exploratory supplementary evaluator to assess interrater consistency with human clinicians; it was not used as a gold standard or a substitute for human expert review. The mean of the 2 clinician scores was used for analysis. Reports were assessed using a 5-point scale across 4 domains: data accuracy: consistency with the original medical report and absence of hallucinated or altered findings; information completeness: inclusion of all clinically relevant abnormalities and important longitudinal changes; appropriateness of interpretation: whether the severity and clinical meaning of findings were explained accurately and with appropriate wording; and reasonableness of recommendations: whether the educational content and general suggestions were medically appropriate. Domain scores and total scores (maximum 20) were compared between clinician evaluation and Kimi evaluation.

### Phase 2: Prospective Clinical Evaluation

In the prospective phase, we enrolled patients undergoing echocardiography in outpatient or inpatient settings between January 2026 and March 2026. Inclusion criteria for patients were (1) age 18 years or older, (2) clear consciousness and ability to complete questionnaire-based assessment, (3) prior hospitalization at our center for acute myocardial infarction or chronic heart failure with echocardiography performed during hospitalization, and (4) undergoing repeat echocardiography at the current visit. For family members, inclusion criteria were (1) age 18 years or older, (2) spouse or first-degree relative of the patient, and (3) willingness to participate. Exclusion criteria were severe cognitive impairment or psychiatric illness, clinical instability, or declined participation. All participants received both the conventional echocardiography report and the patient-friendly report generated by the LLM system.

To ensure clinical safety, we implemented a multilayer risk mitigation strategy: (1) structured input prompts minimizing ambiguity and (2) mandatory clinician review of every generated report before any patient-facing release.

### Patient and Family Questionnaire Assessment

A study-specific 5-point Likert questionnaire (1=strongly disagree, 5=strongly agree) was developed for this study; it has not undergone formal validation, which we acknowledge as a limitation. A study-specific 5-point Likert questionnaire (1=strongly disagree, 5=strongly agree) was used to assess 4 dimensions:

- Helpfulness in understanding report results
- Effectiveness in addressing questions or concerns
- Helpfulness in improving disease-related knowledge
- Degree of anxiety relief

Because not all patients had a corresponding family member and only 85 family members completed the questionnaire, patient and family scores were analyzed as independent samples.

To explore potential differences in perceived benefit, subgroup analyses were performed according to (1) sex, (2) age (>60 y vs ≤60 y), (3) primary diagnosis (acute myocardial infarction vs chronic heart failure), (4) left ventricular ejection fraction (LVEF; >40% vs ≤40%), (5) left ventricular end-diastolic diameter (LVEDD; >55 mm vs ≤55 mm), and (6) care setting (outpatient vs inpatient). All subgroup analyses were exploratory; no adjustment for multiple comparisons was applied, and the findings should be interpreted as hypothesis-generating.

### Anxiety Assessment

Short-term anxiety was assessed using the STAI-6. Raw scores were used, with higher scores indicating greater anxiety. Participants completed the scale anonymously on paper at 3 time points:

- APost-ECHO: after completion of echocardiography (baseline)
- APost-CR: after the release of the conventional report
- APost-PFR: after reading the patient-friendly report

Two change rates (CRs) were calculated:

- CR1 = (APost-CR − APost-ECHO) / APost-ECHO (increase after conventional report)
- CR2 = (APost-CR − APost-PFR) / APost-CR (reduction after patient-friendly report)

### Statistical Analysis

Statistical analyses were performed using SPSS (version 26.0). Continuous variables are presented as mean (SD). Scores from clinicians and Kimi were compared using 2-tailed paired *t* tests. Patient and family questionnaire scores were compared using 2-tailed independent-sample *t* tests. Anxiety scores across the 3 time points were analyzed using repeated-measures ANOVA with post hoc pairwise comparisons. Correlations between CR2 and subjective anxiety relief scores were assessed using Pearson correlation. A two-sided *P*<.05 was considered statistically significant. Because multiple subgroup comparisons were performed without adjustment for multiplicity, all subgroup *P* values are descriptive and should be interpreted cautiously; confirmatory studies are needed.

## Results

### Professional Quality Evaluation of the Generated Reports

In the retrospective phase, the LLM successfully generated patient-friendly reports for all 60 cases, seamlessly integrating longitudinal comparisons with prior examinations. As shown in Table 1, the professional quality of the LLM-generated reports was rated highly across all metrics. The total quality scores (out of a maximum of 20) assigned by the human clinicians (mean 18.15, SD 1.36) and the external LLM evaluator (mean 18.28, SD 1.26) showed high consistency, with no statistically significant difference (*P*=.55). Regarding clinical safety, only 1 instance of AI hallucination was identified among the 60 reports, wherein the LLM incorrectly inferred that the patient required pacemaker therapy based on an isolated conduction abnormality. This finding suggests a low hallucination rate in this small sample but does not establish safety in routine clinical use; larger-scale surveillance is required. This highlights the high baseline accuracy of the prompt framework while underscoring the necessity of human-in-the-loop oversight.

**Table 1.** Professional quality evaluation of the generated patient-friendly echocardiography reports^a^.

Evaluation item	Human clinicians, mean (SD)	Kimi 2.5, mean (SD)	*t* test (*df*)	*P* value
Data accuracy	4.82 (0.54)	4.92 (0.28)	–1.281 (59)	.20
Information completeness	4.77 (0.59)	4.75 (0.60)	0.153 (59)	.88
Appropriateness of interpretation	4.65 (0.66)	4.67 (0.63)	–1.42 (59)	.89
Reasonableness of recommendations	3.92 (0.72)	3.93 (0.71)	–1.28 (59)	.90
Total scores	18.15 (1.36)	18.28 (1.26)	–0.487 (59)	.63

a Report quality was evaluated by 2 clinicians and an external large language model reviewer, Kimi 2.5 (Moonshot AI). Scores ranged from 1 to 5 for each domain, with higher scores indicating better quality.

### Baseline Characteristics and Report Generation in the Prospective Cohort

A total of 100 patients were prospectively enrolled, the mean age was 55.31 (SD 14.44) years, and patient-friendly reports were successfully generated for all participants. The cohort comprised 2 clinical scenarios: chronic heart failure (n=50) and acute myocardial infarction (n=50). In all cases, the first inpatient echocardiography served as the reference study. Baseline characteristics are shown in Table 2.

**Table 2.** Baseline characteristics of patients in the prospective cohort.

Variable	CHF^a^ group (n=50)	AMI^b^ group (n=50)
Age (y), mean (SD)	57.42 (13.55)	53.20 (15.12)
Sex, n (%)		
Male	42 (84)	44 (88)
Female	8 (16)	6 (12)
Comorbidities, n (%)		
Atrial fibrillation	9 (18)	5 (10)
Hypertension	20 (40)	26 (52)
Diabetes mellitus	9 (18)	10 (20)
Chronic renal insufficiency	4 (8)	0 (0)
Echocardiographic findings during hospitalization		
Left ventricular ejection fraction (%), mean (SD)	28.94 (6.20)	44.96 (10.98)
Left ventricular end-diastolic diameter (mm), mean (SD)	65.14 (6.61)	49.36 (7.32)
Moderate-to-severe aortic valve disease, n (%)	4 (8)	2 (4)
Moderate-to-severe mitral valve disease, n (%)	26 (52)	9 (18)
Moderate-to-severe tricuspid valve disease, n (%)	11 (22)	2 (4)
Left ventricular mural thrombus, n (%)	2 (4)	1 (2)

a CHF: chronic heart failure.

b AMI: acute myocardial infarction.

### Patient and Family Evaluation of the Patient-Friendly Reports

Questionnaire responses were obtained from 100 patients and 85 family members. Both groups rated the reports highly, with mean scores above 4.3 across all 4 domains (Table 3). We report these scores descriptively without assigning qualitative labels (eg, “very good”) because interpretative thresholds are arbitrary. Total scores did not differ significantly between patients and family members (17.61, SD 1.60 vs 17.62, SD 1.03; *P*=.95).

**Table 3.** Comparison of patient and family ratings of the patient-friendly echocardiography reports^a^.

Evaluation item	Patients (n=100), mean (SD)	Family members (n=85), mean (SD)	*t* test (*df*)	*P* value
Helpfulness in understanding report results	4.37 (0.49)	4.36 (0.48)	0.74 (183)	.94
Effectiveness in addressing questions or concerns	4.31 (0.84)	4.29 (0.46)	0.156 (183)	.88
Helpfulness in improving disease-related knowledge	4.37 (0.49)	4.38 (0.49)	–0.09 (183)	.93
Degree of anxiety relief	4.56 (0.66)	4.59 (0.50)	–0.326 (183)	.75
Total scores	17.61 (1.60)	17.62 (1.03	–0.067 (183)	.95

a Questionnaire items were scored on a 5-point Likert scale, with higher scores indicating greater perceived benefit. Because only 85 family members completed the questionnaire and not all patients had a corresponding family respondent, comparisons were performed as independent-sample analyses.

### Subgroup Differences in Perceived Benefit

#### Sex

**Figure 2.**
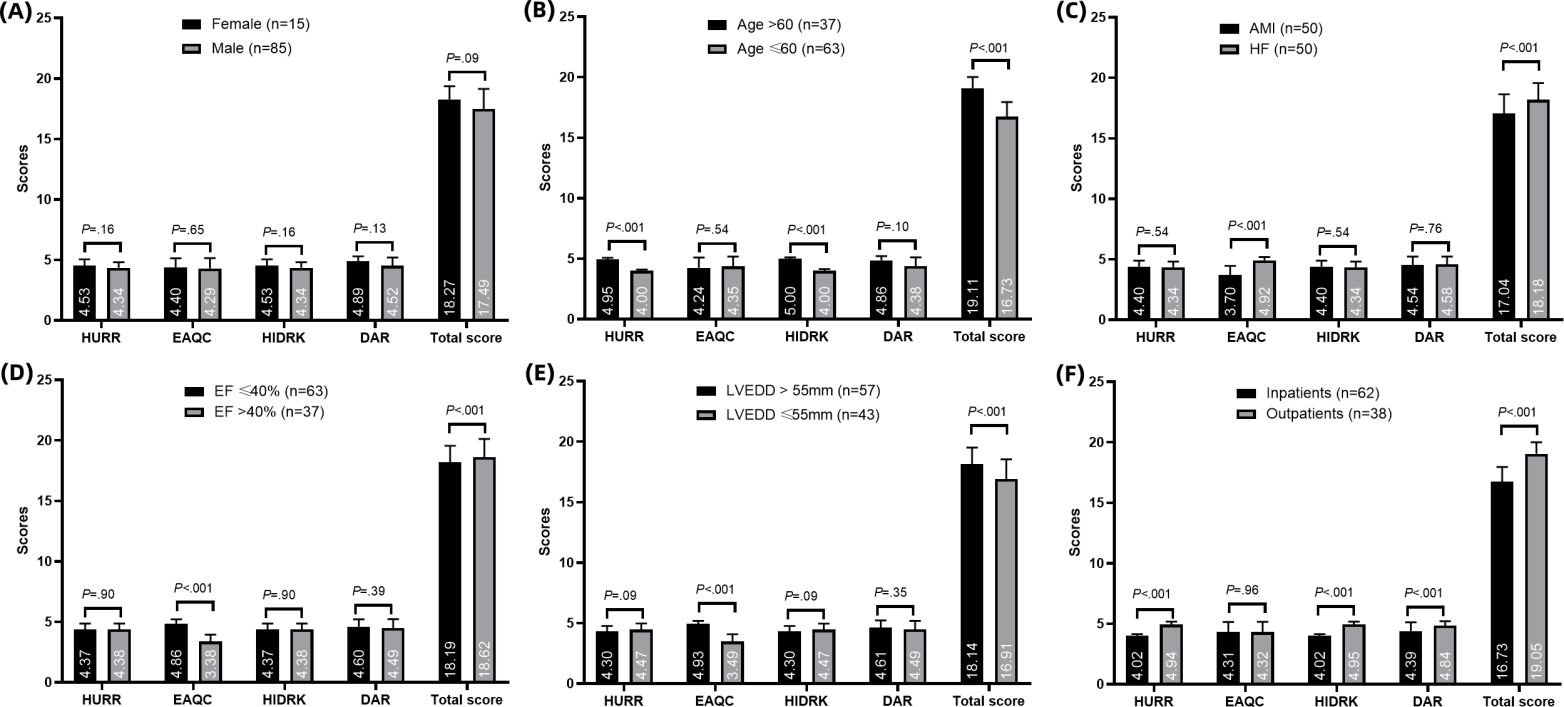
Subgroup analysis of patient ratings of the patient-friendly echocardiography reports. Comparison of questionnaire scores across subgroups defined by sex, age, primary diagnosis, left ventricular ejection fraction (LVEF), left ventricular end-diastolic diameter (LVEDD), and care setting. Higher scores indicate greater perceived benefit from the patient-friendly reports. (A) No significant differences in questionnaire scores were observed by sex. (B) Compared with patients aged 60 years or younger, those older than 60 years reported higher scores in helpfulness in understanding report results (HURR), helpfulness in improving disease-related knowledge (HIDRK), and total scores (*P*<.001). (C) Patients with chronic heart failure (CHF) scored higher than those with acute myocardial infarction (AMI) in effectiveness in addressing questions or concerns (EAQC) and total scores (*P*<.001). (D) Patients with LVEF ≤40% reported higher than those with LVEF>40% in EAQC and total scores (*P*<.001). (E) Patients with LVEDD >55 mm reported higher than those with LVEDD ≤55 mm in EAQC and total scores (*P*<.001). (F) Outpatients reported higher in HURR, HIDRK, degree of anxiety relief (DAR), and total scores (*P*<.001).

As shown in Figure 2, no significant differences in questionnaire scores were observed by sex (Figure 2A).

#### Age

Compared with patients aged 60 years or younger, those older than 60 years reported higher scores for helpfulness in understanding report results (4.95, SD 0.12 vs 4.00, SD 0.11; *P*<.001), helpfulness in improving disease-related knowledge (5.00, SD 0.11 vs 4.00, SD 0.14; *P*<.001), and total scores (19.11, SD 0.91 vs 16.73, SD 1.22; *P*<.001). No significant age-group differences were observed for the other domains (Figure 2B). These age-related findings are exploratory and require confirmation.

#### Primary Diagnosis, LVEF, and LVEDD

Differences were mainly observed in effectiveness when addressing questions or concerns. Patients with chronic heart failure scored higher than those with acute myocardial infarction (4.92, SD 0.27 vs 3.70, SD 0.76; *P*<.001) and also had higher total scores (18.18, SD 1.40 vs 17.04, SD 1.60; *P*<.001). Similarly, patients with LVEF ≤40% reported higher effectiveness in addressing concerns than those with LVEF>40% (4.86, SD 0.35 vs 3.38, SD 0.56; *P*<.001) and also had higher total scores (18.62, SD 1.50 vs 18.19, SD 1.37; *P*<.001). Patients with LVEDD >55 mm scored higher on this domain than those with LVEDD ≤55 mm (4.93, SD 0.26 vs 3.49, SD 0.59; *P*<.001) and also had higher total scores (18.14, SD 1.37 vs 16.91, SD 1.63; *P*<.001; Figure 2C-2E). Again, these subgroup patterns are hypothesis-generating and should be interpreted with caution given the lack of multiplicity adjustment.

#### Care Setting

Outpatients reported greater perceived benefit than inpatients across multiple domains: helpfulness in understanding report results (4.94, SD 0.23 vs 4.02, SD 0.13; *P*<.001), helpfulness in improving disease-related knowledge (4.95, SD 0.23 vs 4.02, SD 0.13; *P*<.001), anxiety relief (4.84, SD 0.37 vs 4.39, SD 0.73; *P*<.001), and total scores (19.05, SD 0.96 vs 16.73, SD 1.23; *P*<.001; Figure 2F).

### Longitudinal Changes in Anxiety

**Figure 3.**
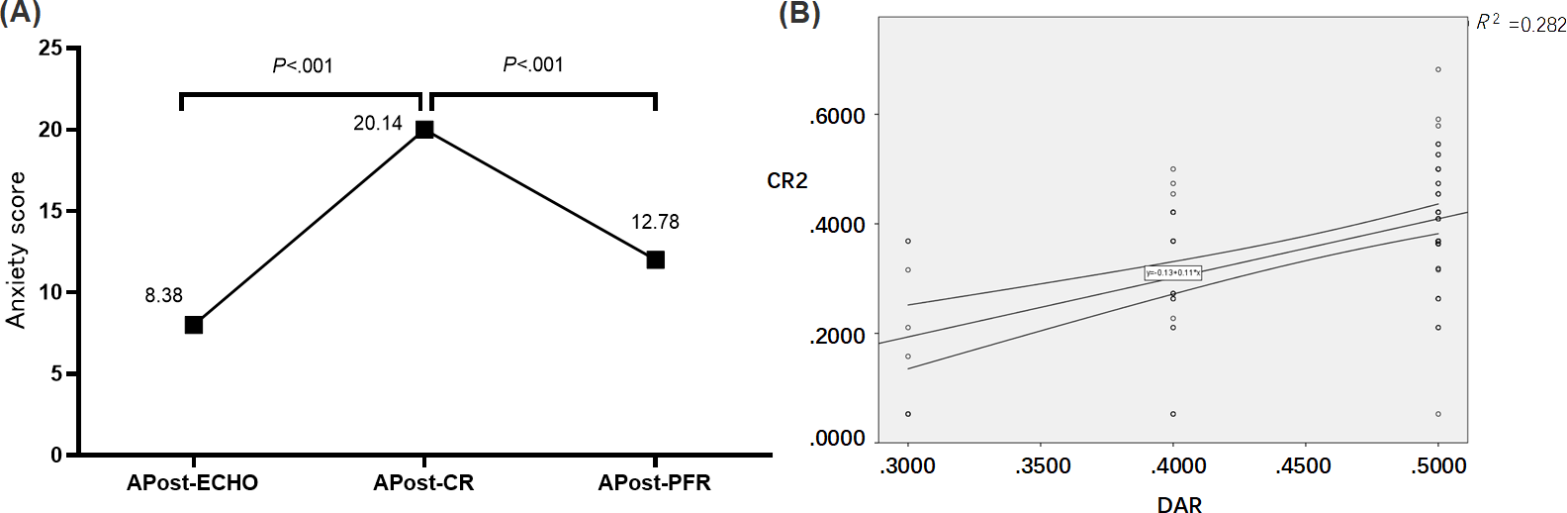
Changes in anxiety scores across the 3 assessment time points. (A) Anxiety was assessed using the raw 6-item short-form State-Trait Anxiety Inventory (STAI-6) score at 3 time points: after echocardiography (APost-ECHO), after the release of the conventional report (APost-CR), and after reading the patient-friendly report (APost-PFR). Higher scores indicate greater anxiety. APost-CR showed a significant increase compared to APost-ECHO (*P*<.001), followed by a significant decrease in APost-PFR compared to APost-CR (*P*<.001). (B) CR2, the anxiety reduction rate after reading the patient‑friendly report, was positively correlated with the subjective anxiety relief ratings (degree of anxiety relief [DAR]) (*r*=0.531; *P*<.001).

Among patients with complete serial assessments, mean STAI-6 raw scores changed significantly across the 3 time points (Figure 3). The mean score after echocardiography (APost-ECHO) was 8.38 (SD 0.49). After the release of the conventional report (APost-CR), the mean score increased to 20.14 (SD 1.46; *P*<.001 vs APost-ECHO). After reading the patient-friendly report (APost-PFR), the mean score decreased to 12.78 (SD 2.42; *P*<.001 vs APost-CR; Figure 3A). We describe these as associations; causality is not established due to the sequential nonrandomized design and potential order effects.

The reduction in anxiety after reading the patient-friendly report (CR2) was positively correlated with the subjective rating of anxiety relief (*r*=0.531; *P*<.001; n=100; Figure 3B).

### Subgroup Analyses of Anxiety Change Rates

**Figure 4.**
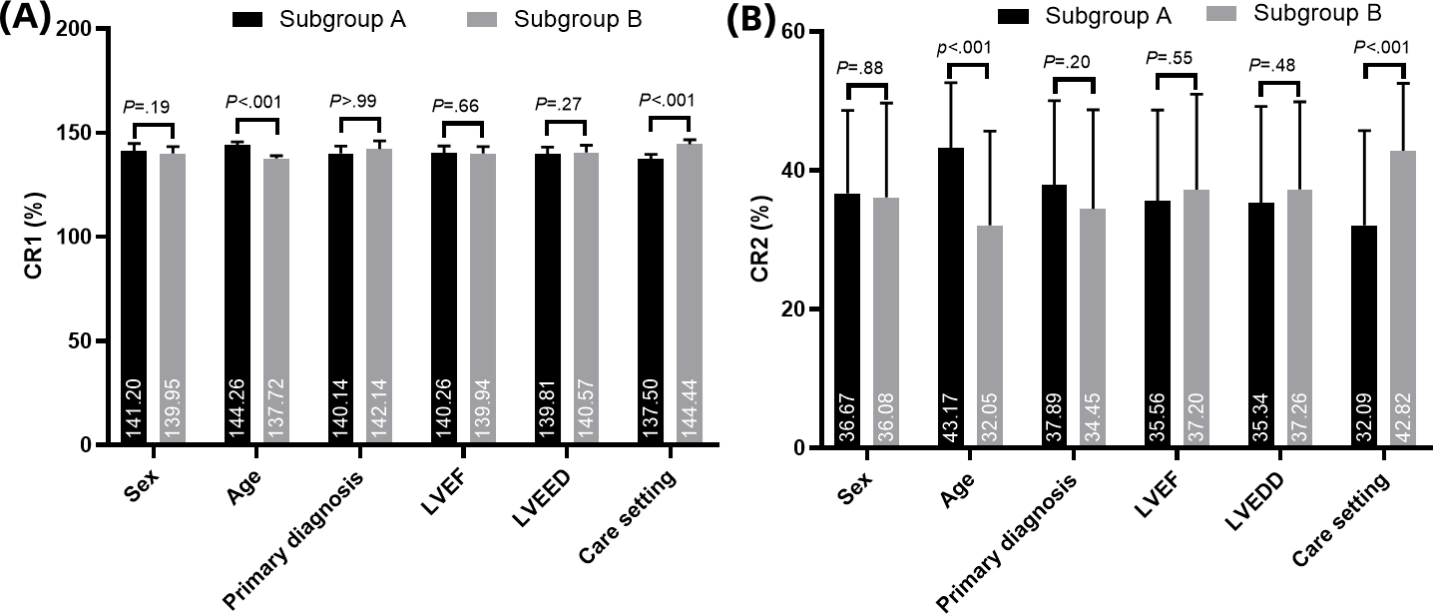
Subgroup analysis of anxiety change rates. Comparison of anxiety change rates across predefined patient subgroups. (A) CR1 was significantly higher in patients older than 60 years and outpatients than in those aged 60 years or younger and inpatients (*P*<.001). (B) A similar significant increase in CR2 was observed in patients older than 60 years and outpatients (*P*<.001). CR1 was calculated as (APost-CR − APost-ECHO) / APost-ECHO, and CR2 was calculated as (APost-CR − APost-PFR) / APost-CR. Higher CR1 indicates a greater increase in anxiety after the release of the conventional report; higher CR2 indicates a greater reduction in anxiety after reading the patient-friendly report. Subgroup definitions: subgroup A includes female, older than 60 years, acute myocardial infarction (AMI), left ventricular ejection fraction (LVEF) ≤40%, left ventricular end-diastolic diameter (LVEDD) >55 mm, and inpatients group. Subgroup B includes male, aged 60 years or younger, chronic heart failure (CHF), LVEF >40%, LVEDD ≤55 mm, and outpatients group. APost-CR: anxiety score after conventional report release; APost-ECHO: anxiety score after echocardiography; APost-PFR: anxiety score after reading the patient-friendly report.

No significant differences in CR1 or CR2 were observed by sex, primary diagnosis, LVEF, or LVEDD. In contrast, patients aged more than 60 years had significantly higher CR1 (144.26%, SD 1.41% vs 137.72%, SD 1.23%; *P*<.001) and higher CR2 (43.17%, SD 9.46% vs 32.05%, SD 13.59%; *P*<.001) than younger patients. Outpatients also had significantly higher CR1 (144.44%, SD 2.23% vs 137.72%, SD 1.23%; *P*<.001) and higher CR2 (42.82%, SD 9.72% vs 32.09%, SD 13.64%; *P*<.001) than inpatients (Figure 4). These exploratory analyses are not adjusted for multiple comparisons and require prospective replication.

## Discussion

### Principal Findings

In this study, we developed and preliminarily evaluated an LLM-based system for generating patient-friendly echocardiography reports with integrated longitudinal comparison. The main findings are 3-fold. First, the generated reports showed preliminary safety signals, with consistently high scores from both clinician reviewers and an external LLM reviewer, and only 1 hallucination event was identified. Second, patients and family members rated the reports favorably across all assessed domains, with particularly high perceived benefit among older adults, outpatients, and patients with chronic heart failure; reduced LVEF (≤40%); or left ventricular enlargement (LVEDD >55 mm). Third, anxiety scores increased significantly after the release of the conventional report but decreased after reading the patient-friendly report, and the magnitude of anxiety reduction was greater in older patients and outpatients. These findings suggest that an LLM-based patient-friendly reporting system with longitudinal comparison is feasible and may provide promising preliminary use for patient and family experience as well as short-term anxiety relief. However, owing to the nonrandomized, sequential design, causality cannot be inferred, and longer-term outcomes remain to be evaluated.

### Comparison With Prior Work

Our findings extend the growing body of literature on LLM applications in patient communication while also acknowledging important risks identified in prior studies, such as hallucinations, lack of longitudinal integration, and limited prospective validation. Previous studies have demonstrated that LLMs can generate readable patient education materials [15], simplify radiology reports [16], and answer patient questions [17,18]. However, several important gaps remain. First, most prior studies have focused on single-examination summaries, lacking longitudinal comparison capability [19]. In cardiovascular care, where disease progression is evaluated through serial imaging, patients need to understand whether their condition has improved, worsened, or remained stable over time [9]. Existing LLM-based report simplification efforts have typically treated each examination in isolation [6,20]. By contrast, our system was explicitly designed to incorporate prior echocardiography reports and clinical background into a unified narrative. Second, most existing studies have been limited to technical feasibility assessments or retrospective analyses, lacking prospective, real-world validation of patient-centered outcomes [21,22]. Our study used a prospective design in a real-world setting, directly collecting questionnaire feedback from 100 patients and 85 family members. Third, our study introduces a novel approach to quantifying psychological impact through multi–time-point anxiety assessment with change rate analysis. While prior research has speculated that patient-friendly reports might reduce anxiety, few studies have empirically measured this effect using validated instruments [23,24]. We assessed anxiety at 3 time points and calculated anxiety change rates. This allowed us to quantify anxiety dynamics and compare changes across subgroups. Taken together, these features—longitudinal comparison, prospective real-world validation, and multi–time-point anxiety measurement with change rate analysis—distinguish our study from prior LLM-based patient communication research. Despite these strengths, our study remains exploratory and nonrandomized, precluding causal claims.

### Professional Quality and Safety Considerations

The professional evaluation results support the feasibility of using LLMs to generate patient-friendly echocardiography reports with a low rate of clinically significant errors in this small sample, but they do not establish safety for routine use. Both clinician reviewers and the external LLM (Kimi) assigned similarly high scores across data accuracy, information completeness, appropriateness of interpretation, and reasonableness of recommendations. This consistency suggests that, when guided by structured inputs and a predefined output format, LLMs can produce summaries that preserve core clinical content while enhancing accessibility for lay readers. However, the identification of one hallucination event—in which the report incorrectly inferred pacemaker treatment—is an important safety consideration. Although infrequent in this cohort, such errors highlight that LLM-generated reports should not be considered fully autonomous outputs in clinical practice. Human oversight remains essential, particularly for statements involving treatment history, disease severity, or management implications [25,26].

Our risk mitigation strategy includes the following measures: (1) failure-mode analysis: we conducted a prestudy failure mode and effects analysis identifying 3 potential failure modes (hallucinated clinical implications, omission of critical longitudinal changes, and misinterpretation of numerical values). For each, we defined detection methods (clinician checklist) and mitigation steps (structured prompt constraints, postgeneration rule-based filters). (2) Systematic error detection: we implemented a continuous error logging system during the retrospective phase; all generated reports were reviewed by 2 independent clinicians using a standardized error taxonomy. The 1 hallucination event was logged and led to a prompt refinement (adding explicit negative constraints). (3) External validation procedures: while full external validation is beyond the scope of this single-center study, we have added a statement that a multicenter external validation (with different LLM backbones and local reporting conventions) is planned as the next step. We also clarify that the current safety conclusions apply only to the specific model version and clinical setting.

Our risk mitigation strategy (structured prompts and mandatory clinician review) likely contributed to the low observed hallucination rate, but ongoing monitoring is required. This finding aligns with prior studies on LLM applications in health care, which have consistently emphasized the need for clinician review and the development of robust quality assurance mechanisms. In our system design, we intentionally structured the input to minimize ambiguity and used a standardized output format, which likely contributed to the low hallucination rate. Nevertheless, ongoing monitoring and refinement are necessary as these models are deployed in clinical settings.

### Patient and Family Experience

The high ratings from both patients and family members across all questionnaire domains indicate that the generated reports were well received by end users. Importantly, no significant differences were observed between patient and family member ratings, suggesting that the reports are equally beneficial for both groups. This is clinically relevant because family members often play an important role in treatment decisions, follow-up planning, and long-term care in cardiovascular disease. A report format that improves understanding not only for patients but also for accompanying family members may help support shared understanding and facilitate more effective communication between families and health care providers.

The subgroup findings, while exploratory, refine our understanding of which populations may perceive greater benefit. Older patients reported greater benefit in report understanding and disease-related knowledge, which may reflect the higher communication barrier posed by conventional technical reports in this population. Health literacy tends to decline with age, and older adults are more likely to struggle with medical terminology and complex numerical data. Outpatients also reported greater benefit than inpatients, possibly because they have less immediate access to bedside explanation and therefore derive greater value from written interpretive support. In contrast, inpatients may receive more real-time explanation from clinicians during hospitalization, reducing their perceived need for written patient-friendly summaries. Together, these findings suggest that the clinical value of patient-friendly reports may be greatest in settings where informational needs are high and routine clinician explanation is limited. These hypotheses require confirmation in adequately powered, prespecified subgroup analyses.

### Anxiety Reduction: Mechanisms and Clinical Implications

One of the most notable findings was the pattern of short-term anxiety change. Anxiety increased substantially after the release of the conventional report and then decreased after reading the patient-friendly report. This pattern suggests that simply providing access to a technically written report may not be sufficient to reassure patients and may instead increase uncertainty when the findings are not readily interpretable. This is consistent with prior research demonstrating that unmet informational needs and lack of comprehension are significant drivers of anxiety in patients undergoing diagnostic testing [27-29].

In contrast, a report that explains the meaning of findings in plain language and places them in longitudinal context may help reduce uncertainty-related distress [30,31]. The positive correlation between CR2 (anxiety reduction after reading the patient-friendly report) and subjective ratings of anxiety relief further supports the consistency between measured change and user-reported benefit. However, because of the sequential design, we cannot rule out that the observed anxiety decrease reflects order effects, habituation, regression to the mean, or expectation bias, so this finding should be interpreted cautiously.

The greater anxiety reduction observed in older patients and outpatients provides additional insight into the populations most likely to benefit from this approach. Older patients may experience larger fluctuations in anxiety because they face greater barriers in interpreting technical written reports due to lower health literacy or reduced familiarity with medical terminology. Similarly, outpatients, who often have less access to bedside explanation and continuous support than inpatients, may experience greater distress when conventional reports are received without immediate clarification. In this context, patient-friendly reports may serve as a supplementary communication tool that helps bridge the gap between test completion and physician consultation.

### Future Directions

Our findings align with and extend recent advances in AI applications in cardiovascular medicine. The patient-friendly echocardiography reports generated by our system demonstrated high professional quality and a low hallucination rate, indicating the feasibility of structured, audit-aware LLM agents previously shown in intensive care unit discharge planning. Consistent with Wang et al [32], this suggests that multistep verification frameworks can be successfully adapted to cross-sectional imaging, even though longitudinal comparison introduces additional clinical complexity. Meanwhile, echoing the superior performance of tree-based models for heart failure prediction from structured laboratory data [33], we reaffirm a key principle: for risk stratification tasks that rely heavily on tabular features (eg, left ventricular ejection fraction, left ventricular end-diastolic diameter, and laboratory values), compact and interpretable models remain robust. Finally, our prospective observations of anxiety reduction illustrate a human-centered human-AI collaboration framework: similar to Gao et al [34], we argue that LLMs can serve as cognitive teammates that enhance patient comprehension while ensuring that ultimate decision-making authority remains with humans. Collectively, these cross-domain findings support the integration of compliance-aware agents, explainable predictors, and human-AI teaming principles to achieve safe, patient-centered digital health interventions.

### Limitations

Several limitations must be acknowledged. First, the prospective phase used a sequential design without a parallel control group or randomization; thus, order effects, expectation bias, and regression to the mean may have influenced anxiety outcomes. Causal claims are not supported. Specifically, we note that expectation effects and other biases inherent to nonrandomized sequential designs may account for a substantial portion of the observed anxiety reduction. We therefore explicitly state that our observed effect (anxiety reduction of ~36% from APost‑CR to APost‑PFR) should be interpreted as an upper‑bound estimate [35]. Second, the study was single-center and conducted in a specific Chinese health care context, limiting generalizability to other health care systems, languages, and cultural settings. In Chinese clinical practice, family members are heavily involved in decision-making; our high family member ratings may not generalize to more individualistic health care cultures where patients act more independently. Our hospital uses a highly structured echocardiography reporting template; institutions with free-text or less standardized reports may see different LLM performance (eg, higher hallucination rates). The LLM was used with Chinese prompts; linguistic nuances (eg, politeness markers, explanatory style) may not translate directly to other languages. Reporting conventions and patient expectations may differ elsewhere. Third, our prospective evaluation focused exclusively on short-term psychological outcomes (acute anxiety immediately postintervention). It remains unknown whether this LLM intervention translates into long-term behavioral changes, such as improved medication adherence, sustained health literacy, or better long-term cardiovascular outcomes. Fourth, the sample size in phase 2, while sufficient to demonstrate significant anxiety reduction, may be underpowered to detect rare adverse psychological reactions or subtle differences across highly specific disease subtypes. Fifth, the patient and family questionnaire was study-specific and not formally validated, which may affect replicability. Sixth, the external LLM (Kimi 2.5) was used as a supplementary evaluator, not a gold standard, and its agreement with human clinicians does not independently validate clinical accuracy. Seventh, we performed multiple subgroup comparisons without adjustment for multiplicity. Although we have reframed all subgroup analyses as exploratory and acknowledged the lack of adjustment for multiple comparisons, the presentation of these results in the main text may still inadvertently suggest a degree of confirmatory evidence. We therefore emphasize that these subgroup findings are strictly hypothesis-generating. They should not be interpreted as definitive or used to guide clinical practice until prospectively validated in adequately powered, preregistered studies with appropriate multiplicity correction. Finally, as LLM capabilities evolve rapidly, the specific performance metrics observed in this study are tied to the model version used at the time of the experiment and may not generalize to future iterations.

### Conclusions

An LLM-based reporting system can, with appropriate safeguards, translate complex, technical echocardiography data—including longitudinal comparisons—into accessible, patient-friendly narratives. In this nonrandomized sequential study, the intervention was associated with bridging the health literacy gap and with reduced acute “scanxiety” triggered by immediate access to raw medical records. This intervention showed particularly promising preliminary usefulness for older adults and outpatients. While a “human-in-the-loop” framework remains essential to safeguard against AI hallucinations, our study provides prospective, real-world evidence that LLMs may enhance patient-centered care and psychological well-being in the era of digital health transparency. Causal confirmation requires randomized controlled trials.

## Abbreviations

AI: artificial intelligence
APost-CR: anxiety score after conventional report release
APost-ECHO: anxiety score after echocardiography
APost-PFR: anxiety score after reading the patient-friendly report
CR: change rate
LLM: large language model
LVEDD: left ventricular end-diastolic diameter
LVEF: left ventricular ejection fraction
STAI-6: 6-item short-form State-Trait Anxiety Inventory
